# An In Vitro Comparison of Zirconia and Hybrid Ceramic Crowns With Heavy Chamfer and Shoulder Finish Lines

**DOI:** 10.7759/cureus.33940

**Published:** 2023-01-18

**Authors:** Priyanka Yadav, Vineet Sharma, Jyoti Paliwal, Kamal K Meena, Rahul Madaan, Balwant Gurjar

**Affiliations:** 1 Prosthodontics, Rajasthan University of Health Sciences (RUHS) College of Dental Sciences, Jaipur, IND; 2 Dentistry, Rajasthan University of Health Sciences (RUHS) College of Dental Sciences, Jaipur, IND; 3 Dentistry, Community Health Center, Jalore, IND

**Keywords:** internal gap, marginal gap, zirconia, hybrid ceramic, finish line configuration

## Abstract

Purpose

This in vitro study aimed to compare the marginal fit and internal adaptation of computer-aided designed and computer-aided manufactured (CAD-CAM) zirconia and hybrid ceramic crowns on heavy chamfer and shoulder finish line designs using silicon replica method.

Materials and methods

Forty die samples were divided into four groups of 10 dies each. Out of 40 diecasts scanned, zirconia crowns were milled on 20 casts (10 prepared with shoulder and 10 prepared with heavy chamfer finish line design), while hybrid ceramic crowns were milled on the rest of the 20 casts. After milling crowns, the silicone replica technique measured the marginal fit and internal adaptation.

Results

The heavy chamfer finish line design provided a better marginal fit than the shoulder finish line design for zirconia and hybrid ceramic crowns. Hybrid ceramic crowns had a better marginal fit and internal adaptation than zirconia crowns, both at heavy chamfer and shoulder finish line design. The gap at the margin was less than the axial and occlusal walls, and the maximum gap was observed in the occlusal area. In addition, the marginal gap was less than the internal gap, which showed a positive correlation with each other.

Conclusion

The study concluded that the difference in CAD-CAM materials and finish line designs influences marginal fit and crown restoration's internal adaptation. A heavy chamfer finish line design provides a better marginal fit for zirconia and hybrid ceramic crowns than a shoulder finish line design. Hybrid ceramic crowns have a better marginal fit and internal adaptation than zirconia crowns in heavy chamfer and shoulder finish lines.

## Introduction

In recent decades, the demand for patients to have an exceptionally natural appearance and metal-free restorations has risen significantly [1]. This has contributed to the development of newer all-ceramic materials that maintain longevity with enhanced mechanical properties [2,3]. As a result, all dental restorations should be esthetically, mechanically, and biologically acceptable [4].

All-ceramic restorations have always had the advantage of aesthetics. The mechanical properties have also improved with yttria-stabilized zirconia as an all-ceramic material and polymer-infiltrated hybrid ceramic material in dentistry. Zirconia has an excellent blend of high flexural strength and fracture toughness. This hybrid ceramic incorporates the advantages of both ceramic and resin in one material [5]. Furthermore, no additive processing steps are needed after milling. All dental restorations should have an excellent marginal fit from a biological point of view as the marginal discrepancy increases the rate of cement dissolution, causing microleakage [6]. This will ultimately lead to pulpitis [7].

The finish line configuration is the crucial aspect that decides the marginal fit. An ideal finish line design results in better marginal fit and allows for the escapement of excess luting cement, resulting in proper restoration seating [8]. An excellent marginal fit will minimize plaque build-up, decreasing recurrent caries and periodontal disease [9]. Internal adaptation has a vital role in the retention and resistance of restoration and plays a positive role in the longevity of the full-coverage restoration [10]. According to Holmes et al., the perpendicular measurement from the internal surface of the casting to the axial wall of the preparation was defined as the internal gap, and the same measurement at the margin was defined as the marginal gap [11].

The marginal fit and internal adaptation of conventionally fabricated all-ceramic restorations are influenced by investing procedures, casting or pressing process, and firing temperatures [12,13]. With the introduction of computer-aided design and computer-aided manufacturing (CAD-CAM) in dentistry, the most significant aspect from the biological point of view, i.e., marginal integrity, could be addressed and controlled. Furthermore, the restoration's marginal fit and internal adaptation could be improved by integrating scanning, designing, and milling.

The methods for measuring the marginal and internal gaps can be broadly divided into two groups. First is the invasive or destructive method (cross-sectioning method to measure the luting film thickness). The second is the non-invasive or non-destructive method (direct viewing, profile projector, microcomputer tomography, digimatic micrometer, and silicone replica technique) [14]. The silicone replica technique is a non-destructive and reliable method to determine the marginal fit and internal adaptation in vivo and in vitro [15].

The present in vitro study compares the marginal fit and internal adaptation of CAD-CAM-fabricated zirconia and hybrid ceramic crowns on heavy chamfer and shoulder finish line designs, keeping in mind the primary goal of prosthetic dentistry, i.e., preservation of the remaining structures.

Research hypothesis

Marginal fit and internal adaptation are independent of CAD-CAM material and finish line design.

## Materials and methods

The present study was conducted in the Department of Prosthodontics, Rajasthan University of Health Sciences (RUHS) College of Dental Sciences, Jaipur, India. It included the following steps.

Tooth preparation and impression-making

**Figure 1:**
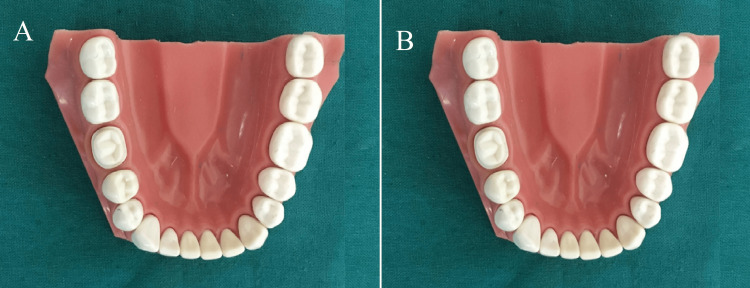
Typodont mandibular right first molar teeth (46) prepared for all-ceramic full-coverage restorations with 1 mm shoulder finish line design (A) and 1 mm heavy chamfer finish line design (B)

**Figure 2:**
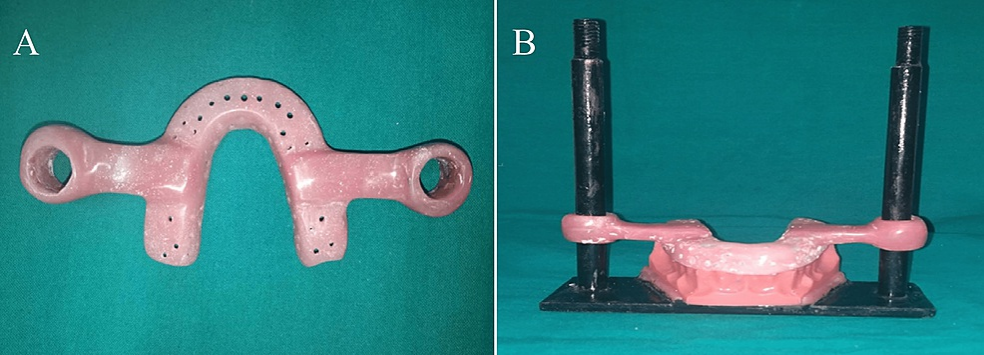
Customized (A) impression tray with horizontal extensions and (B) tray extended horizontally over the vertical extensions of the U-shaped frame for standardized impression

**Figure 3:**
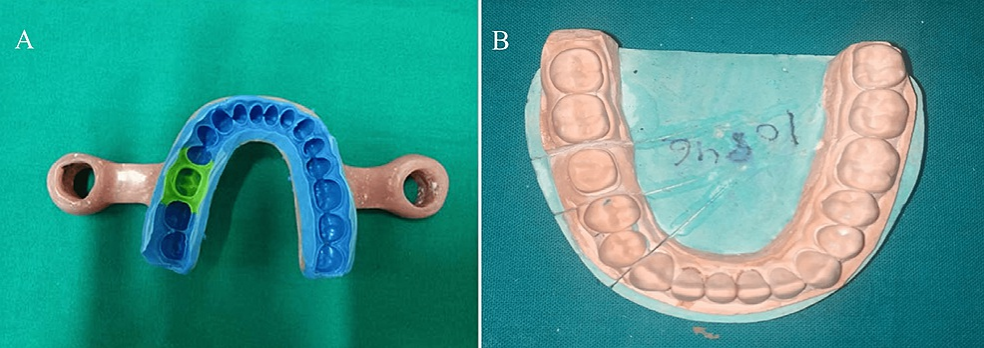
(A) One-step dual viscosity impression and (B) prepared die

According to standard tooth preparation procedures, two typodont (mandibular typodont, Confident Sales India Pvt Ltd., Bangalore, India) mandibular right first molar teeth (46) were prepared for all-ceramic full-coverage restorations, one with a 1 millimeter (mm) shoulder and the other with a 1 mm heavy chamfer (Figure 1). The custom tray was fabricated using auto-polymerizing acrylic resin with 3 mm of uniform space for the impression material and four vertical stops, two on either side. The custom tray was stored for 72 hours to allow for shrinkage during polymerization. The mandibular typodont was fixed to the base of a U-shaped frame with auto-polymerizing acrylic resin. The custom tray was extended horizontally over vertical extensions of the U-shaped frame to ensure a definitive path of seating the custom tray during impression-making using auto-polymerizing acrylic resin (Figure 2). A one-step dual viscosity impression was made under standard room temperature with polyvinyl siloxane (Virtual, Ivoclar Vivadent AG, Schaan, Liechtenstein). First, a heavy body was injected into the custom tray, and at the same time, a light body was injected on and around the prepared tooth. Next, the custom tray assembly was positioned on the typodont set, and pressure was maintained till the material was set. After the impression material was set, the impression was removed and carefully examined for defects (Figure 3).

Die preparation

The impression was poured into a type IV dental stone (Kalrock, Kalabhai Karson Pvt. Ltd., Mumbai, India) and allowed to be set for one hour. Next, the cast was separated from the impression, and the prepared tooth was visually inspected. Then, die-cutting and ditching were done to the prepared tooth. The impression-making and die-preparation processes were repeated 20 times for each prepared tooth. All the procedures, including impression-making and die preparation, were done under standard room conditions.

Grouping of samples

The samples were then divided into four groups of 10 dies each as follows: Group I included CAD-CAM zirconia single crowns with heavy chamfer finish line design, Group II had CAD-CAM zirconia single crowns with shoulder finish line design, Group III had CAD-CAM hybrid ceramic single crowns with heavy chamfer finish line design, and Group IV had CAD-CAM hybrid ceramic single crowns with shoulder finish line design.

Scanning and designing

Each master cast was precisely positioned in the screw jig of the scanner. After scanning the master cast, the die was placed in the die jig and scanned. The three-dimensional image obtained was adjusted and confirmed. The restoration to be fabricated was designed with a 40-micrometer (μm) cement space.

Milling

Out of the 40 casts scanned, zirconia fully anatomic monolithic crowns were milled on 20 casts (10 prepared with shoulder and 10 prepared with heavy chamfer finish line design). Partially sintered zirconia blanks (Cercon ht, Dentsply Sirona Prosthetics, New York, United States) were inserted, and enlarged frameworks were designed and fabricated to compensate for material shrinkage after the final sintering firing. This material was prepared to mill the crown to its total volume as a monolithic material. After milling, the blank was removed, and the objects were then removed from the blank by sandblasting with aluminum oxide with a fine blasting tip. It helps prevent framework fracture or other damage to the objects. The crowns were then separated from the blank, and no adjustments were made to the crowns. The above-mentioned CAD-CAM process was repeated 20 times, and 20 zirconia crowns were milled, 10 with heavy chamfer (Group I) and 10 with shoulder (Group II) finish line design. After milling, all the zirconia crowns were sintered in a closed furnace. Next, 20 hybrid ceramic (VITA ENAMIC, VITA Zahnfabrik, Bad Säckingen, Germany) crowns were milled following the protocol, 10 with heavy chamfer (Group III) and 10 with shoulder (Group IV) finish line design. Next, hybrid ceramic crowns were milled, and no adjustments were made.

Measuring marginal fit and internal adaptation (silicone replica method)

**Figure 4:**
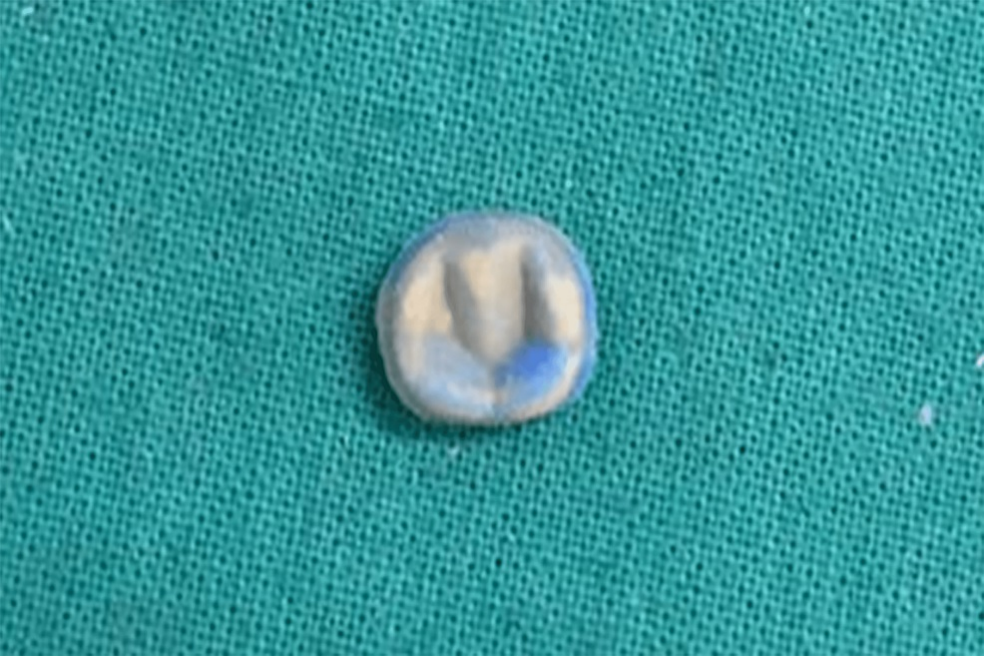
Silicon replica prepared with the light body on the intaglio surface of the crown and a heavy body silicone impression material with a contrasting color

After milling crowns, the silicone replica technique measured the marginal fit and internal adaptation. First, a light body impression material (Virtual, Ivoclar Vivadent AG, Schaan, Liechtenstein) was thoroughly filled in the crown to obtain a replica. Then the crown was seated onto the prepared tooth with a constant load of 50 Newtons using a universal testing machine. After the light body impression material was set, the crown was removed from the tooth along with the thin film of the light body on the intaglio surface of the crown. To support this thin silicon film, a heavy body silicone impression material with a contrasting color was injected into the inner surface of the crown. After the heavy body impression material was set, the excess was cut off, and the single-piece silicone replica was removed (Figure 4).

**Figure 5:**
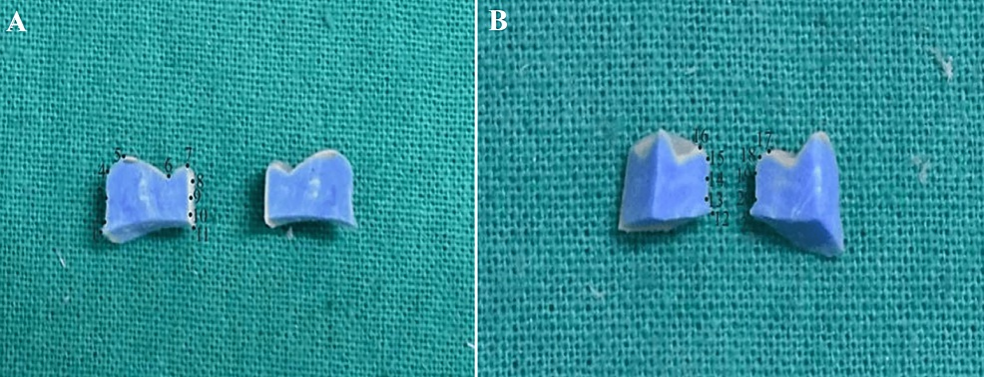
(A) Buccolingual section with reference points and (B) mesiodistal section with reference points

The replica was then sectioned buccolingually and mesiodistally with a Bard Parker blade, and four sections were obtained. Each section sets the four reference points at marginal, marginal-internal, axial, and occlusal. In addition, in the buccolingual section, one more reference point was set at mid-occlusal. Thus, measurements were made at 21 reference points on each silicone replica (Figure 5).

**Figure 6:**
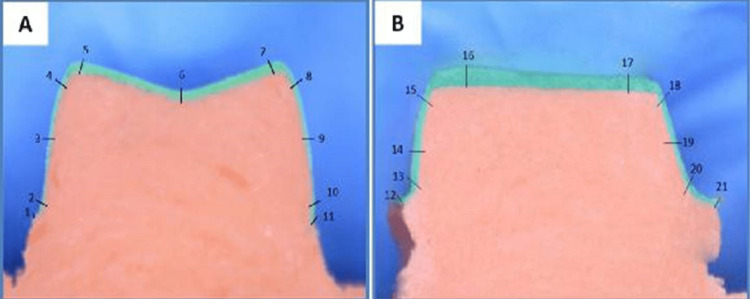
Digital image created under 100x optical microscope: (A) buccolingual section with reference points 1-11 and (B) mesiodistal section with reference points 12-21

The sections were viewed under a 100x optical microscope (ZEISS Axio Imager 2, Carl Zeiss Microscopy Deutschland GmbH, Oberkochen, Germany), and the digital images were analyzed (Figure 6). Measurements of the marginal and internal gaps for the crown were done by measuring the thickness of light body silicone material at 21 predetermined points for each silicone replica. In the same way, marginal fit and internal adaptation were measured for all 40 crowns. Finally, the data obtained were statistically analyzed using the Statistical Package for Social Sciences (SPSS) software. For all the statistical tests, p < 0.05 was considered statistically significant, keeping the α error at 5% and β error at 20%, thus giving power to the study as 80%.

## Results

The mean marginal gap within the 95% confidence interval for Group II (39.41 μm ± 2.731) was the maximum, followed by Group I (31.79 μm ± 1.180), Group IV (25.09 μm ± 0.824), and Group III (22.62 μm ± 1.454). The mean marginal-internal gap for Group II (155.06 μm ± 2.807) was the maximum, followed by Group IV (128.85 μm ± 1.688), Group I (90.08 μm ± 2.516), and Group III (73.19 μm ± 1.990). Group II had the largest mean axial gap (125.01 ± 1.276), followed by Group I (119.03 ± 2.460), Group IV (100.14 m ± 0.809), and Group III (97.82 ± 1.011). The mean occlusal gap for Group II (156.90 μm ± 2.451) was maximum, followed by Group I (154.19 μm ± 2.41), Group IV (128.82 μm ± 1.406), and Group III (116.75 μm ± 1.204) (Tables 1, 2).

**Table 1:** Average value of gaps in all the tested groups

Group	Marginal		Marginal Internal		Axial		Occlusal		Internal		Total	
	Mean Value (μm)	Standard Deviation (μm)	Mean Value (μm)	Standard Deviation (μm)	Mean Value (μm)	Standard Deviation (μm)	Mean Value (μm)	Standard Deviation (μm)	Mean Value (μm)	Standard Deviation (μm)	Mean Value (μm)	Standard Deviation (μm)
I	31.79	1.180	90.08	2.516	119.03	2.460	154.19	64.962	363.30	65.668	395.09	66.002
II	39.41	2.731	155.06	2.807	125.01	1.276	156.90	2.451	436.97	4.029	476.38	5.558
III	22.62	1.454	73.19	1.990	97.82	1.011	116.75	1.204	287.77	2.752	310.39	3.374
IV	25.09	.824	128.85	1.688	100.14	.809	128.82	1.406	357.81	2.524	382.90	2.543
Total	29.73	6.802	111.80	32.594	110.50	11.982	139.17	35.648	361.46	62.135	391.19	67.623

**Table 2:** Pairwise comparison using Tukey's post hoc tests * Statistically significant.

Dependent Variable	Group	Group	Mean Difference (μm)	Standard Error (μm)	Significance
Marginal	I	II	-7.619^*^	.763	.001
	I	III	9.170^*^	.763	.001
	I	IV	6.693^*^	.763	.001
	II	III	16.789^*^	.763	.001
	II	IV	14.312^*^	.763	.001
	III	IV	-2.477^*^	.763	.013
Marginal internal	I	II	-64.977^*^	1.025	.001
	I	III	16.888^*^	1.025	.001
	I	IV	-38.773^*^	1.025	.001
	II	III	81.865^*^	1.025	.001
	II	IV	26.204^*^	1.025	.001
	III	IV	-55.661^*^	1.025	.001
Axial	I	II	-5.976^*^	.684	.001
	I	III	21.212^*^	.684	.001
	I	IV	18.896^*^	.684	.001
	II	III	27.188^*^	.684	.001
	II	IV	24.872^*^	.684	.001
	III	IV	-27.188^*^	.684	.001
Occlusal	I	II	-2.717	14.542	.998
	I	III	37.431	14.542	.065
	I	IV	25.367	14.542	.316
	II	III	40.148^*^	14.542	.043
	II	IV	28.084	14.542	.233
	III	IV	-12.064	14.542	.840
Internal	I	II	-73.671^*^	14.735	.001
	I	III	75.530^*^	14.735	.001
	I	IV	5.490	14.735	.982
	II	III	149.201^*^	14.735	.001
	II	IV	79.160^*^	14.735	.001
	III	IV	-70.041^*^	14.735	.001
Total	I	II	-81.290^*^	14.841	.001
	I	III	84.700^*^	14.841	.001
	I	IV	12.183	14.841	.844
	II	III	165.990^*^	14.841	.001
	II	IV	93.472^*^	14.841	.001
	III	IV	-72.518^*^	14.841	.001

## Discussion

The present study evaluated the influence of different CAD-CAM materials, zirconia, and hybrid ceramic and finish line designs on marginal fit and internal adaptation by the silicone replica method. In dentistry, zirconia has been used as a CAD-CAM biomaterial since 2004 to fabricate crowns and fixed partial dentures. In addition, the partially sintered zirconia has been used to produce a compensating enlarged prosthesis to compensate for shrinkage during sintering [16-19]. Furthermore, hybrid ceramics were introduced in 2013, exhibiting the benefits of ceramic and resin in one material [5].

The present study evaluated marginal, marginal-internal, axial, and occlusal areas to obtain a complete picture of crown seating. The results showed that crowns fabricated with CAD-CAM do not have a homogenous gap along with the tooth preparation, even though a uniform 40 μm cement space was used in designing. This could be attributed to the difference in the quality of acquisition and processing of digital data, the relief of undercut areas, and the diameter and shape of the milling tools to produce fine details. In addition, in CAD-CAM technology, marginal and internal discrepancies arise from over-grinding and chipping thin porcelain margins due to the brittle nature of the material and milling vibration.

In the present study, Group I was compared to Group III and Group II to Group IV to evaluate the material influence on marginal fit and internal adaptation. When the data from restorations fabricated with zirconia was compared to hybrid ceramic, a statistically significant difference was observed in the marginal and internal gaps at both heavy chamfer and shoulder finish line designs. Furthermore, the hybrid ceramic crowns showed fewer marginal and internal gaps for heavy chamfer and shoulder finish line designs. This could be attributed to shrinkage compensation during milling (oversized milled prosthesis) and post-milling heat treatment of zirconia [20].

Compared to hybrid ceramics, which involve wet milling, the process of zirconia was performed in a dry environment. Therefore, a difference in marginal fit and internal adaptation can also occur. Thus, the first research hypothesis that marginal fit and internal adaptation are independent of the CAD-CAM material was rejected.

The present study results showed the maximum internal gap at the occlusal surface. First, the greater occlusal gap could be attributed to the limitations in the scanner resolution, which produces slightly rounded edges. Second, compared to the flat occlusal reduction design used in other studies, it may be due to the planar occlusal reduction for crown preparation. This crown preparation design produces more occlusal gaps due to inaccuracies created during the scanning process based on the "not the same plane surface effect" [21,22]. Finally, a correlation test between the marginal and internal gaps for all four groups showed a positive relationship between the marginal and internal gaps, i.e., when the marginal gap increased, the internal gap also increased as, in each tested group, all areas of the crown (marginal and internal) were exposed to the same milling procedure.

Clinicians have complete control over the finish line design of the preparation since it depends on the preliminary choice of a particular bur shape. At the same time, the values of other variables are less predictable (i.e., the occlusal convergence or the preparation depth). A statistically significant difference was noted in the marginal gap at heavy chamfer and shoulder finish line designs when comparing Group I to Group II and Group III to Group IV. The results showed that the marginal gap of crowns at the heavy chamfer finish line design was lower than that at the shoulder finish line design. The heavy chamfer finish line design enables a more accurate crown seat through the easy removal of excess luting cement than the shoulder finish line design, which may lead to incomplete crown seating, thereby increasing the vertical marginal gap. The precision of scanner detection is also affected by variations in preparation depth. Another potential source is the restricted accuracy of the milling process, which results in an increased variety in the depth of preparation. These findings support the superiority of heavy chamfer finish line design and are inconsistent with the study by Pera et al. [23]. Hence, the second research hypothesis that the marginal fit and internal adaptation are independent of the finish line design was also rejected.

On comparing Group I to Group IV, the mean difference was 6.693. The mean marginal gap of hybrid ceramic crowns at the shoulder finish line design is 25.09 μm, and the mean marginal gap of zirconia crowns at the heavy chamfer finish line is 31.79 μm. This indicates that the choice of CAD-CAM material significantly influences marginal fit and internal adaptation of crowns more than the finish line design.

The marginal gap is considered the most important to evaluate all the assessment measures from a clinical perspective. McLean and Christenson [24,25] found a clinically acceptable marginal gap of less than 120 μm. In the present study, both zirconia and hybrid ceramic crowns had a mean marginal value of less than 43 μm at both heavy chamfer and shoulder finish line design. The clinically acceptable limit for the axial gap is 122 μm [26]. All tested groups in the present study have an axial gap of 97.82-125.01 μm, which is within the clinically acceptable limit. In addition, all four tested groups have occlusal gap values ranging from 116.75 to 156.90 μm, which is within the clinically acceptable limit as has been supported by the study by Karlsson [27]. Similarly, Bindl and Mörmann found the internal gap in 49-136 μm [28]. All the tested groups have internal gap values ranging from 95.92 to 145.66 μm, which is within the clinically acceptable limit.

In the present study, quantitative analysis of the marginal and internal gaps was done using the optical microscope, giving two-dimensional data. Three-dimensional measuring with computerized techniques may provide more accurate results. Hence, one limitation of this study was that only a limited number of data points could be assessed by sectioning and measuring the silicone replica samples using an optical microscope. Also, the defects of replicas could prevent the correct adaptation of the restorations from being measured. Another limitation of this study is that the marginal and internal gaps were measured with crowns on their respective prepared typodont teeth without cementing them. The pressure of the cement layer might change the results of the measurements obtained in this study due to the difference in the physical properties of the luting cement and the light body polyvinyl siloxane used in this study. Therefore, long-term clinical studies should be carried out to update the clinically acceptable limit regarding marginal fit and internal adaptation.

## Conclusions

The study has predicted that the difference in CAD-CAM materials and finish line designs influences the marginal fit and crown restorations' internal adaptation. A heavy chamfer finish line design provides a better marginal fit for zirconia and hybrid ceramic crowns than a shoulder finish line design. Hybrid ceramic crowns have a better marginal fit and internal adaptation than zirconia crowns in heavy chamfer and shoulder finish lines. The gap at the margin is less than that at the axial and occlusal walls, and the occlusal area has the greatest gap. The marginal gap is less than the internal gap, with a positive correlation between the marginal and internal gaps. This indicates that any crown restoration with poor marginal fit will also have poor internal adaptation. Both zirconia and hybrid ceramic crowns have a marginal and internal gap within the clinically acceptable limit at the shoulder and heavy chamfer finish line design.

## References

[1] Zarone F, Ferrari M, Mangano FG, Leone R, Sorrentino R (2016). "Digitally Oriented Materials": Focus on Lithium Disilicate Ceramics. Int J Dent.

[2] Denry I, Kelly JR (2008). State of the art of zirconia for dental applications. Dent Mater.

[3] Zarone F, Russo S, Sorrentino R (2011). From porcelain-fused-to-metal to zirconia: clinical and experimental considerations. Dent Mater.

[4] Boening KW, Wolf BH, Schmidt AE, Kästner K, Walter MH (2000). Clinical fit of Procera AllCeram crowns. J Prosthet Dent.

[5] Awada A, Nathanson D (2015). Mechanical properties of resin-ceramic CAD/CAM restorative materials. J Prosthet Dent.

[6] Jacobs MS, Windeler AS (1991). An investigation of dental luting cement solubility as a function of the marginal gap. J Prosthet Dent.

[7] Goldman M, Laosonthorn P, White RR (1992). Microleakage---full crowns and the dental pulp. J Endod.

[8] Gavelis JR, Morency JD, Riley ED, Sozio RB (2004). The effect of various finish line preparations on the marginal seal and occlusal seat of full crown preparations. 1981. J Prosthet Dent.

[9] Felton DA, Kanoy BE, Bayne SC, Wirthman GP (1991). Effect of in vivo crown margin discrepancies on periodontal health. J Prosthet Dent.

[10] Svanborg P, Skjerven H, Carlsson P, Eliasson A, Karlsson S, Ortorp A (2014). Marginal and internal fit of cobalt-chromium fixed dental prostheses generated from digital and conventional impressions. Int J Dent.

[11] Holmes JR, Bayne SC, Holland GA, Sulik WD (1989). Considerations in measurement of marginal fit. J Prosthet Dent.

[12] Mously HA, Finkelman M, Zandparsa R, Hirayama H (2014). Marginal and internal adaptation of ceramic crown restorations fabricated with CAD/CAM technology and the heat-press technique. J Prosthet Dent.

[13] Ng J, Ruse D, Wyatt C (2014). A comparison of the marginal fit of crowns fabricated with digital and conventional methods. J Prosthet Dent.

[14] Anadioti E, Aquilino SA, Gratton DG, Holloway JA, Denry IL, Thomas GW, Qian F (2015). Internal fit of pressed and computer-aided design/computer-aided manufacturing ceramic crowns made from digital and conventional impressions. J Prosthet Dent.

[15] Nawafleh NA, Mack F, Evans J, Mackay J, Hatamleh MM (2013). Accuracy and reliability of methods to measure marginal adaptation of crowns and FDPs: a literature review. J Prosthodont.

[16] Bona AD, Pecho OE, Alessandretti R (2015). Zirconia as a dental biomaterial. Materials (Basel).

[17] Silva NR, Sailer I, Zhang Y, Coelho PG, Guess PC, Zembic A, Kohal RJ (2010). Performance of zirconia for dental healthcare. Materials (Basel).

[18] Yus EA, Cantarell JM, Alonso AM (2018). Comparison of the marginal fit of milled yttrium stabilized zirconium dioxide crowns obtained by scanning silicone impressions and by scanning stone replicas. J Adv Prosthodont.

[19] Rajan BN, Jayaraman S, Kandhasamy B, Rajakumaran I (2015). Evaluation of marginal fit and internal adaptation of zirconia copings fabricated by two CAD - CAM systems: An in vitro study. J Indian Prosthodont Soc.

[20] Abduo J, Lyons K, Bennamoun M (2014). Trends in computer-aided manufacturing in prosthodontics: a review of the available streams. Int J Dent.

[21] Borba M, Miranda WG Jr, Cesar PF, Griggs JA, Bona AD (2013). Evaluation of the adaptation of zirconia-based fixed partial dentures using micro-CT technology. Braz Oral Res.

[22] Colpani JT, Borba M, Della Bona A (2013). Evaluation of marginal and internal fit of ceramic crown copings. Dent Mater.

[23] Pera P, Gilodi S, Bassi F, Carossa S (1994). In vitro marginal adaptation of alumina porcelain ceramic crowns. J Prosthet Dent.

[24] McLean JW, von Fraunhofer JA (1971). The estimation of cement film thickness by an in vivo technique. Br Dent J.

[25] Christensen GJ (1966). Marginal fit of gold inlay castings. J Prosthet Dent.

[26] Tuntiprawon M, Wilson PR (1995). The effect of cement thickness on the fracture strength of all-ceramic crowns. Aust Dent J.

[27] Karlsson S (1993). The fit of Procera titanium crowns. An in vitro and clinical study. Acta Odontol Scand.

[28] Bindl A, Mörmann WH (2005). Marginal and internal fit of all-ceramic CAD/CAM crown-copings on chamfer preparations. J Oral Rehabil.

